# Antiphospholipid antibodies levels and potential effects on in-vitro fertilization in a large cohort of infertile Syrian women

**DOI:** 10.1016/j.amsu.2021.102301

**Published:** 2021-04-18

**Authors:** Haya Deeb, Omar Abdul Salam, Venus Shaaban, Adnan Alkhatib, Nawras Alhalabi, Marwan Alhalabi

**Affiliations:** a Faculty of Medicine, Damascus University, Damascus, Syria; b Al-Khatib Laboratory, Damascus, Syria; c Faculty of Medicine, Syrian Private University, Damascus, Syria; d Assisted Reproductive Unit, Oriental Hospital, Damascus, Syria; e Department of Reproductive Medicine, Embryology and Genetics, Faculty of Medicine, Damascus, Syria

**Keywords:** Antiphospholipid antibodies, Antiphospholipid syndrome, Anticardiolipin antibodies, Lupus anticoagulation, In vitro fertilization, Infertility

## Abstract

**Background:**

Obstetric morbidities represent a common hallmark manifestation of antiphospholipid syndrome (APS), with the recurrent loss of pregnancy as the main complication. The presence of antiphospholipid antibodies (APA) and its potential impact have not been established yet in infertile women seeking assisted reproduction technologies in Syria.

This study aims to determine the prevalence of anticardiolipin antibodies (aCL) and lupus anticoagulation (LAC) and their association with age and the In vitro fertilization (IVF) outcomes in a large sample of Syrian women.

**Materials and methods:**

The electronic patients’ records were screened and relevant data extraction was performed retrospectively. The study included 876 women who had IVF between January 2012 and January 2020 in a tertiary care hospital.

**Results:**

The prevalence of APA among the included women was less than 1%. Only 5 patients had positive APA. No correlation was found between the APA levels and age. Additionally, the APA did not have an impact on neither the IVF outcomes nor the number of IVF cycles.

**Conclusion:**

the added value of APS antibodies’ screening could be considered very modest when compared to its financial burden on patients since it has a very low prevalence in women having IVF.

## Highlights

• Obstetric morbidities are a hallmark manifestation of antiphospholipid syndrome.
• The prevalence of APS in Syrian women having IVF was less than 1%.
• No correlation was found between the APA levels and age.
• The APA had no impact on the IVF outcomes or the number of IVF cycles.
• Screening of APA constitutes a financial burden on patients more than its benefit.

## 1 Background

Antiphospholipid syndrome (APS) is an autoimmune disorder characterized by the presence of anti-phospholipid antibodies –especially anticardiolipin and lupus anticoagulant-with or without vascular thrombosis and pregnancy morbidity [1].

APS can occur primarily in patients without any clinical or laboratory evidence of other diseases, or it can be associated with other disorders like systematic lupus erythematous (SLE) [2]. or rarely with infections [3], malignancies [4], and the use of certain drugs [1].

Obstetric morbidities represent a common hallmark manifestation of APS, with recurrent pregnancy loss as the main complication. It can also cause placental insufficiency, pre-eclampsia and late fetal death [5]. Several pathogenic mechanisms have been suggested about the effects of APS in obstetrical morbidities. Intraplacental thrombosis, maternal–fetal blood exchange impairment, acute or chronic inflammation, necrosis, and villous infracts are histological features in the placenta of APS patients [6].

Recently, various contradicting studies have demonstrated the prevalence of APS between different populations: 1–5% in general population, 6% of patients with pregnancy morbidities [7], 22% in infertile women [8] and 30–34% in women having in-vitro fertilization (IVF) [8,9]. However, the exact prevalence is not yet identified. Furthermore, the correlation between IVF outcomes and the presence of APA in women having IVF is of great important in clinical practice. Some researchers had suggested a positive relationship between the presence of APA and IVF outcomes [10]. On the other hand, many other studies excluded the correlation [11,12]. Because of those contradictory results, this subject needs further studying, as the effect of APA on women having IVF is not yet established.

In this study, we aim to determine the prevalence of anticardiolipin antibodies (aCL) IgM, IgG and lupus anticoagulant (LAC) in a sample of Syrian women having IVF cycles, and to evaluate the correlation of APA with age. Furthermore, the effects of APA on the IVF outcomes and the number of IVF cycles were also studied.

## 2 Materials and Methods

### 2.1 Data collection

We retrospectively studied the data of all patients who underwent IVF at the Oriental-Hospital in Damascus, Syria between January 2012, and January 2020 were retrospectively studied, all of which gave their consent for their medical files to be used in clinical research. The ethical approval of this retrospective cohort study was obtained from an independent Ethical and Research Committees in The Ethical committee at the Faculty of Medicine, Damascu University, with the approval of Orient. The ethical approval was conducted according to the principles of the Declaration of Helsinki. The study is consistent with the STROCSS guidelines [13], and it is available on Research Registry with a unique registration number: researchregistry6483 (https://www.researchregistry.com/browse-the-registry#home/registrationdetails/600c01351e46090020de44f2/).

The electronic patients' records were reviewed retrospectively and data regarding patients’ demographics, history of IVF, anti-bodies laboratory values, numbers of retrieved oocytes, IVF technique and the conception occurrence after oocytes transplantation were collected.

All patients, that had IVF and a screening test for APA: Lupus Anticoagulation (LAC) antiCardiolipin (aCL) IgM and antiCardiolipin (aCL) IgG, were included in our study. Additionally, in patients who had recurrence failure of the IVF cycles during the study period, only their last IVF outcomes were included in the study due to missing data in their medical history. All the cases that were treated with heparin or aspirin due to previously diagnosed SLE or APS or any other diseases were excluded. Patients that had a complication associated with IVF were also excluded because the IVF was terminated medically so the effects of APS on IVF could not be determined. A total of 876 patients met the previously mentioned criteria and were categorized into three groups based on their age.

### 2.2 Antibodies assay

Before the IVF-treatment, patients’ blood samples were collected and analyzed at the same laboratory. Anticardiolipin antibodies IgG, IgM were tested using enzyme-linked immunosorbent assay (ELISA) by using Aesku Diagnostics© kits. Based on the laboratory criteria of the international preliminary classification [14]: the anticardiolipin antibodies IgG, IgM were considered positive >40 U/ml. However, Lupus anticoagulant was assayed by a three-step procedure, the details of this assay were previously described [15].

### 2.3 IVF protocol

All women, that underwent IVF, had been treated by using long Gonadotropin-releasing hormone (GnRH) agonist or GnRh antagonist for pituitary suppression. The details of those protocols were previously discussed and described [[16], [17], [18]]. Then the patients received human menopausal gonadotropin (HMG), or recombinant follicular stimulating hormone (rFSH), or both, to stimulate follicular growth. Human chorionic gonadotrophin (HCG) was administered after an ultrasound documentation of follicular maturity along with the blood level of estradiol. When 3 leading follicles reached 17–18 mm, 10000 IU of HCG were administered and then after 34–36 h, the transvaginal ultrasound-guided oocytes retrieval was performed. Intra cytoplasmic sperm injection was done for fertilization. In cleavage stage (day 3), three embryos were transferred to the uterine cavity with the transvaginal ultrasound guidance. At the end, the IVF outcomes were measured after two weeks of the embryo transfer and were considered positive by positive urine HCG tests and transvaginal ultra-sonographic evidence of a gestational sac. And the outcomes were considered as a failure when no pregnancy or evidence of a gestational sac had been detected.

### 2.4 Statistical analysis

Only women that met the aforementioned criteria were included in the study. The including patient were sub-grouped by age, IVF outcome, and IVF recurrence. The statistical analysis was made using the Statistical Package for Social Sciences (Version 25; SPSS Inc., Chivcago, IL, USA). Whether the results were normally distributed or not, they were expressed as mean ± standard deviation (SD) or as mean ± standard error of the mean (SEM), respectively. And the prevalence of the syndrome was expressed as percentage. One-way ANOVA Test, Chi-Square and Fisher's exact test were used where appropriate. P value less than 0.05 was considered as a significant statistical result.

## 3 Results

Between January 2012 and January 2020, a total of 876 patients attended The Oriental-Hospital for IVF and met the inclusion criteria of the study. The patients were categorized into three groups based on their age (Table 1). The mean age of the sample was 32.57 ± 6.112 (mean ± sd, range: 17–48).

**Table 1:** Age comparison between the patients’ groups.

	Group 1: age ≤25	Group 2: age [26–35]	Group 3: age ≥ 36
Frequency (percentage)	110 (12.6%)	479 (54.7%)	287 (32.8%)
Age (mean ± SD)	22.59 ± 2.25	30.73 ± 2.79	39.47 ± 2.75

The mean number of oocytes obtained after stimulation for the IVF was 12.99 ± 8.857 (mean ± sd, range: 1–66). When comparing the mean of the retrieved oocytes count (Table 2), there was a significant difference between the means of the three groups (p < 0.05); which suggested that the numbers of oocytes decreased with the increasing of age.

**Table 2:** Comparing by One-way ANOVA between the means of Oocytes, IgM and IgG antibodies between the three groups.

Variables	Group 1: ge ≤ 25	Group 2: age [26–35]	Group 3: age ≥ 36	P-value
Oocytes^a^	18.39 ± 9.98	14.19 ± 8.80	8.91 ± 6.51	0.000
aCL IgM^b^	2.41	2.45	2.41	NS^c^
aCL IgG^b^	3.59	3.98	3.98	NS^c^

a Mean ± SD.

b Mean.

c Not Significant.

The mean of anticardiolipin antibodies (IgM) was 2.430 ± 0.07 (Mean ± SEM, range: 1–40.2). The mean of each age category is mentioned in Table 2. Based on the criteria that we mentioned in the material and methods section, there was only one patient who met the criteria of positive aCL IgM (Table 3). As for, the mean of anticardiolipin antibodies IgG was 3.932 ± 0.17 (Mean ± SEM, range: 1.6–140). The mean of each group is mentioned in Table 2. There are only 3 patients with positive aCL IgG, and 2 patients with positive Lupus anticoagulant antibodies, one of them had also had a positive aCL IgG (Table 3).

**Table 3:** Demographic of the patients with positive APL antibodies.

Patient	Age	aCL IgM	aCL IgG	LAC	IVF recurrence	IVF outcomes
1	40	8.6	52.5 (Positive)	Positive	1	F
2	43	7.9	43.7(Positive)	Negative	2	F
3	30	1.9	130 (Positive)	Negative	1	P
4	26	40.2(Positive)	18.6	Negative	4	F
5	39	4.1	4.3	Positive	1	F
Total^a^		1/876 (0.11%)	3/876 (0.34%)	2/876 (0.23%)		
Total of APA positive^a^ = 5/876 (0.57%)						

a Frequency (percentage).

In comparing the means of aCL IgM and IgG between the three age groups, there was no significant association the antibodies and age (p-value was >0.05) (Table 2).

Based on our findings, 5 patients out of 876 patients had positive antiphospholipid antibodies (aCL IgM or IgG or lupus anticoagulant), suggesting that the prevalence of the presence of these antibodies in women undergoing IVF in Damascus is less than 1% (Table 3).

By studying the association between the antibodies results (negative or positive) with the IVF cycles’ results, there was no association between the tested antibodies and the IVF outcomes (Table 4).

**Table 4:** The association between the IVF outcomes and APA by using Chi-square test/Fisher's exact test.

Variables		IVF outcomes		P-value
		Failed	Positive	
aCL IgM	Positive	1	0	NS^a^
	Negative	480	395	
aCL IgG	Positive	2	1	NS^a^
	Negative	479	394	
LAC	Positive	2	0	NS^a^
	Negative	479	395	

a Not Significant.

Moreover, there was no association between the APA results and the numbers of recurrence IVF cycles (Table 5), in other words the APA seem to have no impact on the recurrence failure of IVF.

**Table 5:** The association between APA and numbers of IVF by using Chi-square test/Fisher's exact test.

Variables		Number of IVF cycles		P-value
		First IVF	Recurrence IVF	
aCL IgM	Positive	0	1	NS^a^
	Negative	695	180	
aCL IgG	Positive	3	0	NS^a^
	Negative	692	181	
LAC	Positive	2	0	NS^a^
	Negative	693	181	

a Not Significant.

## 4 Discussion

To the best of our knowledge, this is the first study in Syria that investigated the prevalence of APS antibodies in a large sample of Syrian women having IVF.

In this retrospective study, the prevalence of APS antibodies was 0.57%. This result is relatively low compared to previous cohort studies: a retrospective study was 5.9% [11], and a prospective study was 7.76% [19]; although other studies reported a very high prevalence rate of 32.6%–39.6% [12,20]. Additionally, the prevalence of each antibody (aCL IgM, IgG and LAC) is very low compared to other reports [11,21]. One of the studies in the field did not detect any patients with positive LAC but there was only one positive patient with aCL IgG [12]. Another study had not found any women with positive anticardiolipin IgG antibody [19]. This disparity in the prevalence of APA may be due to the differences in patients’ inclusion criteria, or in the numbers and types of the antibodies tested. Also, the study type of the comparable researches and their cohort size may be one of the factors that affect the results.

Even though the number of patients having IVF cycles was large, this does not demonstrate the actual number of infertile women in need of an IVF cycles due to the high cost of IVF compared to the national average income. This reason could be a major limitation in our study, and it may explain the very low number of cases with positive APA, and its approximation to APA prevalence of the general health population [7]. Another reason might be the absence of screening standards and considering this test a routine test in women with history of infertility.

While our study did not manage to assess the association between APA and IVF outcomes, other previous studies reported no association between them [11,12], while others provided contradictory results [10]. This difference may be due to several factors including: type of study, racial differences or even due to the sample size and the inclusion criteria.

Furthermore, the antibody levels had shown no significant association with age. This result is consistent with previous findings [12]. But this absence of correlation does not reduce the importance of detecting the positivity of APA especially among women older than 35, as there is a significant inverse relationship between age and number of oocytes obtained in IVF cycle, which can increase the chance of having good-quality embryos replaced [22].

APS does not seem to affect the overall IVF outcomes in our hospital due to unavailability of enough data and the APA decreased prevalence. There were other limitations in this study: the inability to obtain sufficient data about patients, such as the cause of infertility and medical history.

Based on the previous findings; the low prevalence of APA and the absence of their association with age, IVF cycles’ outcomes and the number of IVF recurrence suggest that testing for APS antibodies constitutes a financial burden on patients that exceeds its clinical benefit. So we highly recommend to not using this test as routine test in women undergoing IVF cycles unless they have clinical features of APS we previously discussed. However, more future researches on Syrian infertile women are needed to study the effects of APA on IVF outcomes with more demographic information about patients like abortions and clinical features of the syndrome.

## 5 Conclusion

The correlation between APS antibodies and the IVF cycles’ outcome is of great interest to obstetricians and gynecologists in their clinical practice. Our study showed a very low prevalence of APS antibodies in women having IVF. Furthermore, we found that the presence of APA is not correlated with age or the number of recurrent failure IVF cycles. Therefore, screening the APS antibodies constitutes a financial burden on patients more than its benefit.

## Funding

There were no sources of funding for the research.

## Availability of data and material

The datasets, that was used and analyzed during the current study, are available from the corresponding author on reasonable request.

## Ethics approval and consent to participate

The ethical approval was obtained from an independent Ethical and Research Committees in The Oriental Hospital, Damascus, Syria.

## Consent for publication

Not applicable.

## Declaration of competing interest

None.

## Authors’ contributions

All the authors contributed in the study concept and design. MA and AA clinically approached the patients. HA, OA and VS retrospectively checked the patients’ files. HA and NA conducted the statistical analysis. HA, OA and VS drafted the manuscript. NA, MA and AA revised the manuscript for scientific accuracy. All the authors revised the final draft of the manuscript and approved it for publication.

## Provenance and peer review

Not commissioned, externally peer-reviewed.

## List of abbreviations

APS: AnitPhospholipid Syndrome
APA: AnitPhospholipid Antibodies
aCL: antiCardiolipin
LAC: Lupus Anticoagulation
IVF: In Vitro Fertilization
SLE: Systematic Lupus Erythematous
ELISA: Enzyme-Linked ImmunoSorbent Assay
GnRH: Gonadotropin-Releasing Hormone
HMG: Human Menopausal Gonadotropin
rFSH: recombinant Follicular Stimulating Hormone
HCG: Human Chorionic Gonadotropin
IU: International Unit
