# Supplementary material for: Antiphospholipid antibodies levels and potential effects on in-vitro fertilization in a large cohort of infertile Syrian women
Source: Ann Med Surg (Lond). 2021 Apr 18;65:102301. doi: 10.1016/j.amsu.2021.102301 (PMC8079963; doi:10.1016/j.amsu.2021.102301)
Supplement: Multimedia component 1 [file mmc1.docx]

| The STROCSS 2019 Guideline | | |
| --- | --- | --- |
| Item no. | **Item description** | **Page** |
| TITLE | | |
| 1 | Title:   - The word cohort or cross-sectional or case-controlled is included - The area of focus is described (e.g. disease, exposure/intervention, outcome) - Key elements of study design are stated (e.g. retrospective or prospective) | 1 |
| ABSTRACT | | |
| 2a | Introduction: the following points are briefly described   - Background - Scientific Rationale for this study | 1 |
| 2b | Methods: the following areas are briefly described   - Study design (cohort, retro-/prospective, single/multi-centred) - Patient populations and/or groups, including control group, if applicable - Interventions (type, operators, recipients, timeframes) - Outcome measures | 1 |
| 2c | Results: the following areas are briefly described   - Summary data (with statistical relevance) with qualitative descriptions, where appropriate | 1 |
| 2d | Conclusion: the following areas are briefly described   - Key conclusions - Implications to practice - Direction of and need for future research | 1 |
| INTRODUCTION | | |
| 3 | Introduction: the following areas are described in full   - Relevant background and scientific rationale - Aims and objectives - Research question and hypotheses, where appropriate | 1,2 |
| METHODS | | |
| 4a | Registration and ethics   - Research Registry number is stated, in accordance with the declaration of Helsinki* - All studies (including retrospective) should be registered before submission   *"*Every research study involving human subjects must be registered in a publicly accessible database before recruitment of the first subject*" (this can be obtained from: ResearchRegistry.com or ClinicalTrials.gov or ISRCTN) | 2 |
| 4b | Ethical Approval: the following areas are described in full   - Necessity for ethical approval - Ethical approval, with relevant judgement reference from ethics committees - Where ethics was unnecessary, reasons are provided | 2 |
| 4c | Protocol: the following areas are described comprehensively   - Protocol (*a priori* or otherwise) details, with access directions - If published, journal mentioned with the reference provided | 2,3 |
| 4d | Patient Involvement in Research   - Describe how, if at all, patients were involved in study design e.g. were they involved on the study steering committee, did they provide input on outcome selection, etc. | 2,3 |
| 5a | Study Design: the following areas are described comprehensively   - ‘Cohort’ study is mentioned - Design (e.g. retro-/prospective, single/multi-centred) | 2 |
| 5b | Setting: the following areas are described comprehensively   - Geographical location - Nature of institution (e.g. academic/community, public/private) - Dates (recruitment, exposure, follow-up, data collection) | 2,3 |
| 5c | Cohort Groups: the following areas are described in full   - Number of groups - Division of intervention between groups | 2,3 |
| 5d | Subgroup Analysis: the following areas are described comprehensively   - Planned subgroup analyses - Methods used to examine subgroups and their interactions | 3 |
| 6a | Participants: the following areas are described comprehensively   - Eligibility criteria - Recruitment sources - Length and methods of follow-up | 2,3 |
| 6b | Recruitment: the following areas are described comprehensively   - Methods of recruitment to each patient group - Period of recruitment | 2,3 |
| 6c | Sample Size: the following areas are described comprehensively   - Margin of error calculation - Analysis to determine study population - Power calculations, where appropriate | 2,3 |
| INTERVENTION AND CONSIDERATIONS | | |
| 7a | Pre-intervention Considerations: the following areas are described comprehensively   - Patient optimisation (pre-surgical measures) - Pre-intervention treatment (hypothermia/-volaemia/-tension; ICU care; bleeding problems; medications) | 3 (antibodies test) |
| 7b | Intervention: the following areas are described comprehensively   - Type of intervention and reasoning (e.g. pharmacological, surgical, physiotherapy, psychological) - Aim of intervention (preventative/therapeutic) - Concurrent treatments (antibiotics, analgaesia, anti-emetics, NBM, VTE prophylaxis) - Manufacturer and model details where applicable | 3 (IVF procedure) |
| 7c | Intra-Intervention Considerations: the following areas are described comprehensively   - Administration of intervention (location, surgical details, anaesthetic, positioning, equipment needed, preparation, devices, sutures, operative time) - Pharmacological therapies include formulation, dosages, routes and durations - Figures and other media are used to illustrate | 3 |
| 7d | Operator Details: the following areas are described comprehensively   - Training needed - Learning curve for technique - Specialisation and relevant training | - |
| 7e | Quality Control: the following areas are described comprehensively   - Measures taken to reduce variation - Measures taken to ensure quality and consistency in intervention delivery | - |
| 7f | Post-Intervention Considerations: the following areas are described comprehensively   - Post-operative instructions and care - Follow-up measures - Future surveillance requirements (e.g. imaging, blood tests) | 3 |
| 8 | Outcomes: the following areas are described comprehensively   - Primary outcomes, including validation, where applicable - Definitions of outcomes - Secondary outcomes, where appropriate - Follow-up period for outcome assessment, divided by group | 3 |
| 9 | Statistics: the following areas are described comprehensively   - Statistical tests, packages/software used, and interpretation of significance - Confounders and their control, if known - Analysis approach (e.g. intention to treat/per protocol) - Sub-group analysis, if any | 3 |
| RESULTS | | |
| 10a | Participants: the following areas are described comprehensively   - Flow of participants (recruitment, non-participation, cross-over and withdrawal, with reasons) - Population demographics (prognostic features, relevant socioeconomic features, and significant numerical differences) | 4 |
| 10b | Participant Comparison: the following areas are described comprehensively   - Table comparing demographics included - Differences, with statistical relevance - Any group matching, with methods | 4,5,6 |
| 10c | Intervention: the following areas are described comprehensively   - Changes to interventions, with rationale and diagram, if appropriate - Learning required for interventions - Degree of novelty for intervention | 5,6 |
| 11a | Outcomes: the following areas are described comprehensively   - Clinician-assessed and patient-reported outcomes for each group - Relevant photographs and imaging are desirable - Confounders to outcomes and which are adjusted | 4,5,6 |
| 11b | Tolerance: the following areas are described comprehensively   - Assessment of tolerance - Loss to follow up, with reasons (percentage and fraction) - Cross-over with explanation | 5,6 |
| 11c | Complications: the following areas are described comprehensively   - Adverse events described - Classified according to Clavien-Dindo classification* - Mitigation for adverse events (blood loss, wound care, revision surgery should be specified)   *Dindo D, Demartines N, Clavien P-A. Classification of Surgical Complications. A New Proposal with Evaluation in a Cohort of 6336 Patients and Results of a Survey. Ann Surg. 2004; 240(2): 205-213 | - |
| 12 | Key Results: the following areas are described comprehensively   - Key results, including relevant raw data - Statistical analyses with significance | 4,5,6 |
| DISCUSSION | | |
| 13 | Discussion: the following areas are described comprehensively   - Conclusions and rationale - Reference to relevant literature - Implications to clinical practice - Comparison to current gold standard of care - Relevant hypothesis generation | 6,7 |
| 14 | Strengths and Limitations: the following areas are described comprehensively   - Strengths of the study - Limitations and potential impact on results - Assessment of bias and management | 6,7 |
| 15 | Implications and Relevance: the following areas are described comprehensively   - Relevance of findings and potential implications to clinical practice are detailed - Future research that is needed is described, with study designs detailed | 7 |
| CONCLUSION | | |
| 16 | Conclusions:   - Key conclusions are summarised - Key directions for future research are summarised | 7,8 |
| DECLARATIONS | | |
| 17a | Conflicts of interest   - Conflicts of interest, if any, are described | 9 |
| 17b | Funding   - Sources of funding (e.g. grant details), if any, are clearly stated | 8 |
